# Triage Performance Across Large Language Models, ChatGPT, and Untrained Doctors in Emergency Medicine: Comparative Study

**DOI:** 10.2196/53297

**Published:** 2024-06-14

**Authors:** Lars Masanneck, Linea Schmidt, Antonia Seifert, Tristan Kölsche, Niklas Huntemann, Robin Jansen, Mohammed Mehsin, Michael Bernhard, Sven G Meuth, Lennert Böhm, Marc Pawlitzki

**Affiliations:** 1 Department of Neurology Medical Faculty and University Hospital Düsseldorf Heinrich Heine University Düsseldorf Düsseldorf Germany; 2 Digital Health Center Hasso Plattner Institute University of Potsdam Potsdam Germany; 3 Emergency Department Medical Faculty and University Hospital Düsseldorf Heinrich Heine University Düsseldorf Düsseldorf Germany

**Keywords:** emergency medicine, triage, artificial intelligence, large language models, ChatGPT, untrained doctors, doctor, doctors, comparative study, digital health, personnel, staff, cohort, Germany, German

## Abstract

**Background:**

Large language models (LLMs) have demonstrated impressive performances in various medical domains, prompting an exploration of their potential utility within the high-demand setting of emergency department (ED) triage. This study evaluated the triage proficiency of different LLMs and ChatGPT, an LLM-based chatbot, compared to professionally trained ED staff and untrained personnel. We further explored whether LLM responses could guide untrained staff in effective triage.

**Objective:**

This study aimed to assess the efficacy of LLMs and the associated product ChatGPT in ED triage compared to personnel of varying training status and to investigate if the models’ responses can enhance the triage proficiency of untrained personnel.

**Methods:**

A total of 124 anonymized case vignettes were triaged by untrained doctors; different versions of currently available LLMs; ChatGPT; and professionally trained raters, who subsequently agreed on a consensus set according to the Manchester Triage System (MTS). The prototypical vignettes were adapted from cases at a tertiary ED in Germany. The main outcome was the level of agreement between raters’ MTS level assignments, measured via quadratic-weighted Cohen κ. The extent of over- and undertriage was also determined. Notably, instances of ChatGPT were prompted using zero-shot approaches without extensive background information on the MTS. The tested LLMs included raw GPT-4, Llama 3 70B, Gemini 1.5, and Mixtral 8x7b.

**Results:**

GPT-4–based ChatGPT and untrained doctors showed substantial agreement with the consensus triage of professional raters (κ=mean 0.67, SD 0.037 and κ=mean 0.68, SD 0.056, respectively), significantly exceeding the performance of GPT-3.5–based ChatGPT (κ=mean 0.54, SD 0.024; *P*<.001). When untrained doctors used this LLM for second-opinion triage, there was a slight but statistically insignificant performance increase (κ=mean 0.70, SD 0.047; *P*=.97). Other tested LLMs performed similar to or worse than GPT-4–based ChatGPT or showed odd triaging behavior with the used parameters. LLMs and ChatGPT models tended toward overtriage, whereas untrained doctors undertriaged.

**Conclusions:**

While LLMs and the LLM-based product ChatGPT do not yet match professionally trained raters, their best models’ triage proficiency equals that of untrained ED doctors. In its current form, LLMs or ChatGPT thus did not demonstrate gold-standard performance in ED triage and, in the setting of this study, failed to significantly improve untrained doctors’ triage when used as decision support. Notable performance enhancements in newer LLM versions over older ones hint at future improvements with further technological development and specific training.

## Introduction

In recent years, machine learning techniques have been integrated into various aspects of medical care, serving as supportive diagnostic algorithms in clinical applications that include electrocardiograms [1], skin lesion classification [2], and radiological imaging [3]. With the recent boom of generative artificial intelligence (AI) and large language models (LLMs), thrust into the spotlight by the release of ChatGPT (Open AI) [4] in November 2022, the application of generative AI methods has sparked widespread discussion and experimentation in the medical domain.

To many medical professionals, it is remarkable that systems such as ChatGPT without any specialized training have been able to pass US medical exams [5,6]; provide answers to questions from a web-based platform that are preferred over doctors’ replies [7]; and in most cases, offer appropriate recommendations to questions on cardiovascular disease prevention [8]. The capabilities of text generation and summarization introduce numerous controversial research and clinical use cases such as creating discharge summaries [9] or crafting scientific abstracts [10]. Recently, even commercial solutions for automated documentation of clinician-patient interactions have become available [11,12]. All these use cases carry the promise of improving quality and efficiency by offering second opinions [13] and reducing the documentation burden on strained health care professionals [12].

Emergency departments (EDs) frequently serve as the initial point of contact for patients in need of immediate medical attention. A crucial component of ED operations is the triage process, which aims to efficiently allocate often limited resources by prioritizing patients according to the severity and urgency of their conditions. This is typically done using established triage systems, such as the Manchester Triage System (MTS), which is popular in Europe and frequently used in Germany [14,15]. Taking place in a high-stress environment [16,17], triage processes have been shown to be highly variable in quality [18] and influenced by personal traits of the rater such as experience and triaging fatigue [19].

In light of the challenges faced by staff undertaking the triage process and the demonstrated medical abilities of language models, our study sought to assess the capability and potential of ChatGPT in the context of emergency triage. We evaluated its performance in triaging patient vignettes according to the MTS framework, comparing its results to those of both professional MTS raters and doctors working in an ED without triage training. Given the promising data of LLMs serving as a second opinion in other medical contexts [13], we explored ChatGPT’s potential as a resource for providing external validation and second opinions to ED staff with less experience and without specific training in the MTS. We further compared ChatGPT and doctor triages to triage assessments of other currently available state-of-the-art LLMs such as Gemini 1.5 (Google) [20], Llama 3 70B (Meta) [21], and Mixtral 8x7b (Mixtral AI) [22]. Additionally, we tested the performance of the LLM GPT-4 (accessed via the OpenAI application programming interface), on which the product ChatGPT is based on. Our research is the first to compare rater expertise and different LLMs and ChatGPT versions while incorporating the MTS, building on findings of fair agreement between professional raters and Generative Pre-trained Transformer (GPT)–based models in smaller samples using a different triage system [23] and similarities observed between the triage decisions of ophthalmology trainees and GPT-4–based ChatGPT [24].

## Methods

### Case Vignette Creation and Triage Process

In this study, we compiled 124 independent emergency cases from a single, randomly selected day in the interdisciplinary ED of University Hospital Düsseldorf, Germany, which were then transformed into anonymized English case vignettes. These vignettes solely contained medically relevant information, extracted by 1 doctor (LM) according to a predefined standard operating procedure. Patient ages were randomly adjusted within a range of –2 and +2 years where they were not pertinent to the case. Nonmedical information, including personal demographics such as race, was excluded. Vital signs or information about the conscious state of the patient were added where available or deemed clinically necessary. Clinical values were slightly altered (up to 5% of the original value) or added where necessary for triage, with all adjustments made under the discretion of the overseeing doctor. Detailed information on laboratory test results or imaging were not included in case descriptions. The resulting case vignettes (see Textbox S1 in Multimedia Appendix 1) were then reviewed by a second doctor (MP) to ensure the absence of potentially identifying data, in accordance with an internal standard operating procedure. Both doctors were not involved in the subsequent rating of the cases. The cases were independently assessed by 2 MTS-instructing and experienced staff members. When differing triage priorities were assigned, a third equally qualified and MTS-trained doctor mediated the discussion and had a tiebreaking vote to reach a consensus rating (consensus set). Triage decisions were made using the fifth version of the MTS (German version) [25], which consists of the following categories: MTS Level 1 (“Red”) for immediate assessment, MTS Level 2 (“Orange”) for very urgent assessment within 10 minutes, MTS Level 3 (“Yellow”) for urgent assessment within 30 minutes, MTS Level 4 (“Green”) for standard assessment within 90 minutes, and MTS Level 5 (“Blue”) for nonurgent assessment within 120 minutes. The distribution of the determined triage levels for the analyzed day were compared to long-term averages of the ED.

**Figure 1:**
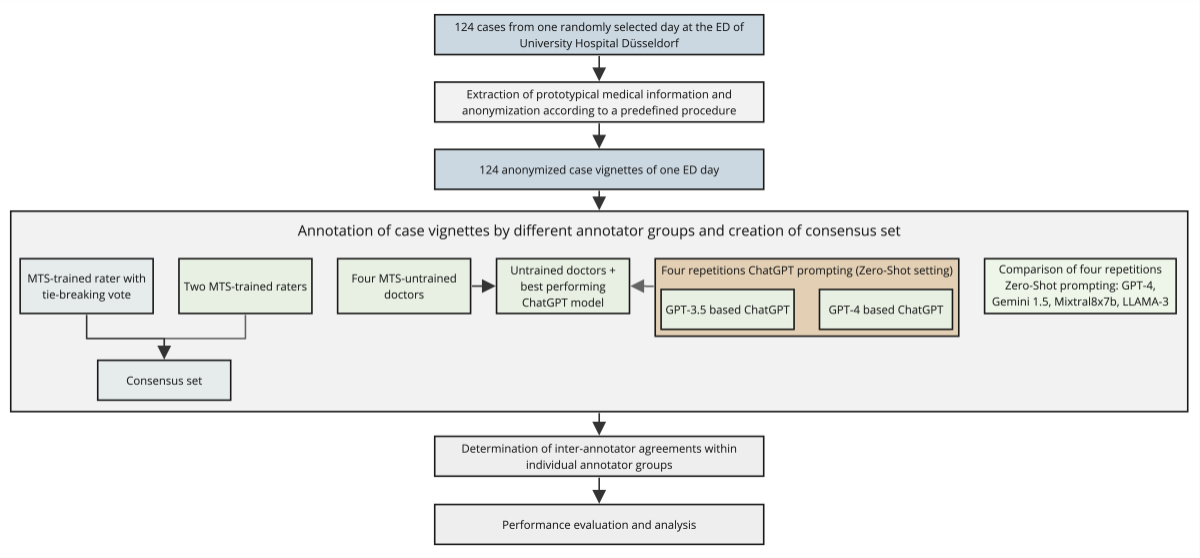
Summarizing flowchart of the study setup. The displayed flowchart summarizes the methodological approach and gives an overview of emergency department (ED) case vignette creation and triage performance assessment. Triage was carried out according to the Manchester Triage System (MTS) by doctors of different training levels and different Generative Pre-trained Transformer (GPT)–based versions of ChatGPT, as well as other large language models (LLMs).

Additionally, 4 MTS-untrained resident doctors regularly working in the ED were given these cases to assess according to MTS triage categories, albeit without access to the precise algorithm diagrams underlying the MTS. These 4 doctors all had no formal MTS training and worked in the ED regularly throughout their residency, with 2 in the second year of residency and 2 in the third year of residency. We also presented the prototypical anonymized case vignettes to 2 versions of ChatGPT, based on either GPT-3.5 or GPT-4 (ChatGPT version: May 24, 2023). The prompt given to ChatGPT was derived after manually testing several prompt versions, with the most promising one selected for the final evaluation in a zero-shot setting. The models were tasked with stratifying the cases according to the MTS using this optimized prompt without further training or refinement (see Textbox S2 in Multimedia Appendix 1) and without access to the copyright-protected MTS algorithm diagrams. Both versions of ChatGPT were queried identically 4 times with new chats for each iteration, which was done due to the probabilistic nature of LLMs [26]. Afterward, the untrained doctors were presented with the answers (rating and explanation) from the overall best-performing ChatGPT instance as a second opinion and were asked to reconsider their initial answers (see Textbox S3 in Multimedia Appendix 1). A “hypothetical best” performance in this scenario was defined as the optimal integration of assistant doctors’ decisions with ChatGPT responses, representing the maximum possible improvement achievable with ChatGPT input. For comparison, a variety of state-of-the-art LLMs (GPT-4, Llama 3 70B, Gemini 1.5, and Mixtral 8x7b) were similarly queried 4 times via respective application programming interfaces in a zero-shot approach using a slightly adapted prompt (see Textbox S2 in Multimedia Appendix 1). Details regarding the models and parameters are described in Table S1 in Multimedia Appendix 1. A flowchart of the study’s setup can be found in Figure 1.

### Calculation of Interrater Agreement

For calculating the interrater agreement, we computed the quadratic-weighted Cohen κ for each rater against the consensus set and used qualitative categories according to the original work by Cohen [27]. To test for statistical differences between the κ values of different rating groups, we performed a 1-way ANOVA with a Bonferroni correction, followed by the Tukey honest significant difference test. Mean values are displayed with SDs throughout the paper. All calculations were executed using Python (version 3.8; Python Software Foundation) employing the *statsmodel* package (version 0.13.2) [28]. For designing graphical representations, we used the *matplotlib* (version 3.1.3) [29] and *seaborn* (version 0.12.0) [30] packages.

### Ethical Considerations

The local ethics committee of University Hospital Düsseldorf (5794R and 2020-1263) granted approval for data collection. Given the retrospective nature of this study, the requirement for written informed consent was waived. Data were anonymized as explained above and no compensation was provided.

## Results

The consensus triage distribution in our analyzed exemplary cases was similar to that of the triage from the same ED in 2022 or published 2019 data [31]. However, there was a higher proportion of patients triaged as “Blue” (9/124, 7.3% vs 4.5% of all cases in 2022), “Yellow” (52/124, 41.9% vs 34.4%), “Orange” (24/124, 19.4% vs 9.5%), and “Red” (5/124, 4% vs 3%). Conversely, fewer patients were triaged as “Green” (34/124, 27.4% vs 48.7%).

**Figure 2:**
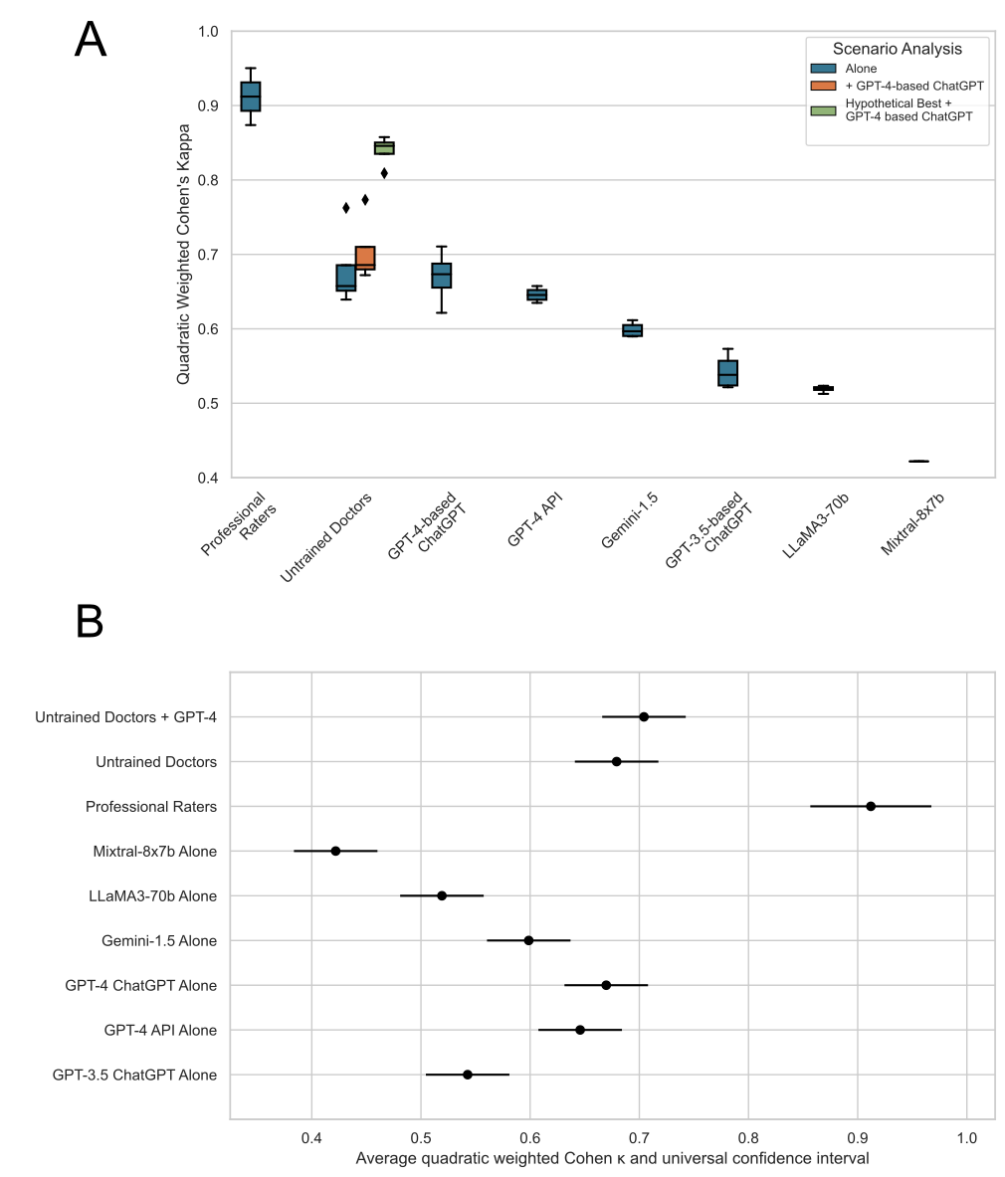
Quadratic-weighted Cohen κ compared to the consensus set. (A) This panel shows box plots of the quadratic-weighted Cohen κ for various triaging groups in relation to the consensus triage (blue). For the untrained doctors' group, both scenarios are depicted: rating alone (initial triage in blue) and rating with GPT-4 as a second opinion (orange). The green box plot represents the potential best combination of the doctors' initial triage with the GPT-4–based ChatGPT second opinion. The box plot's center line indicates the median, box limits indicate upper and lower quartiles, and whiskers indicate 1.5x the IQR. The black point indicates an outlier. (B) This panel illustrates the results of the Tukey honest significant difference test, performed based on an ANOVA with Bonferroni correction of the quadratic-weighted Cohen κ values for the different rater groups. The universal CIs of each group's mean were computed based on Tukey Q and plotted, with nonoverlapping intervals indicating statistically significant differences. As the professional raters' ratings were taken into account for the consensus set, their performance serves primarily as the reference point. API: application programming interface.

As observed in the study, the different versions of ChatGPT and other LLMs were generally capable of providing triage colors as requested using the prompt and consequently assigned an MTS color for each case vignette (see Figures S1 and S2 in Multimedia Appendix 1 for a graphical depiction of all ratings of all raters). We found moderate to substantial agreement between the consensus triage and the triages made by various groups, including LLMs, different ChatGPT iterations, and untrained doctors. As might be anticipated, the professionally trained raters individually exhibited near-perfect alignment with the consensus set they helped construct before its formation, reflected by an average quadratic-weighted κ of 0.91 (SD 0.054). The interrater agreement of the professional raters (quadratic-weighted κ of 0.82) was identical to that reported in the literature [32]. On the other hand, GPT-3.5–based ChatGPT demonstrated only moderate agreement with the consensus set (κ=mean 0.54, SD 0.024), a score that was significantly lower than those of both GPT-4–based ChatGPT (κ=mean 0.67, SD 0.037; *P*<.001) and untrained doctors (κ=mean 0.68, SD 0.056; *P*<.001). The latter 2 groups displayed substantial agreement with the consensus triage, with no significant difference discernible between them (*P*>.99). When the untrained doctors were given GPT-4–based ChatGPT responses as a second opinion, they achieved a slightly higher, although not statistically significant, average κ of 0.70 (SD 0.047; *P*=.97). Despite this improvement, their κ scores still fell short of the hypothetical best combinations of the GPT-4–based ChatGPT responses and the untrained doctors’ initial assessments. Such combinations could have led to near-perfect agreement (κ=mean 0.84, SD 0.021)—a level of performance more comparable to that of professional raters. The other tested LLMs showed varying results, with raw GPT-4 performing very similarly to the GPT-4–based ChatGPT (κ=mean 0.65, SD 0.010; *P*=.98). Gemini 1.5 achieved results that fell between GPT-4 and the GPT-3.5–based ChatGPT with a mean κ of 0.60 (SD 0.010), which were not significantly different from both models (*P*=.08 and *P*=.29, respectively) but significantly worse than untrained doctors (*P*=.03). Llama 3 70B achieved a similar albeit slightly lower average κ of 0.52 (SD 0.004) than the GPT-3.5–based ChatGPT (*P*=.98), which was worse than Gemini 1.5 (*P*=.04) but better than the Mixtral 8x7b model, which had a mean κ of 0.42 (SD 0.000; *P*=.006; see Figure 2; for the respective tables and *P* values [ANOVA: *F*_8_=54.01; *P*<.001], see Table S2 in Multimedia Appendix 1). The κ values of individual raters are shown in Figure S3 in Multimedia Appendix 1.

**Figure 3:**
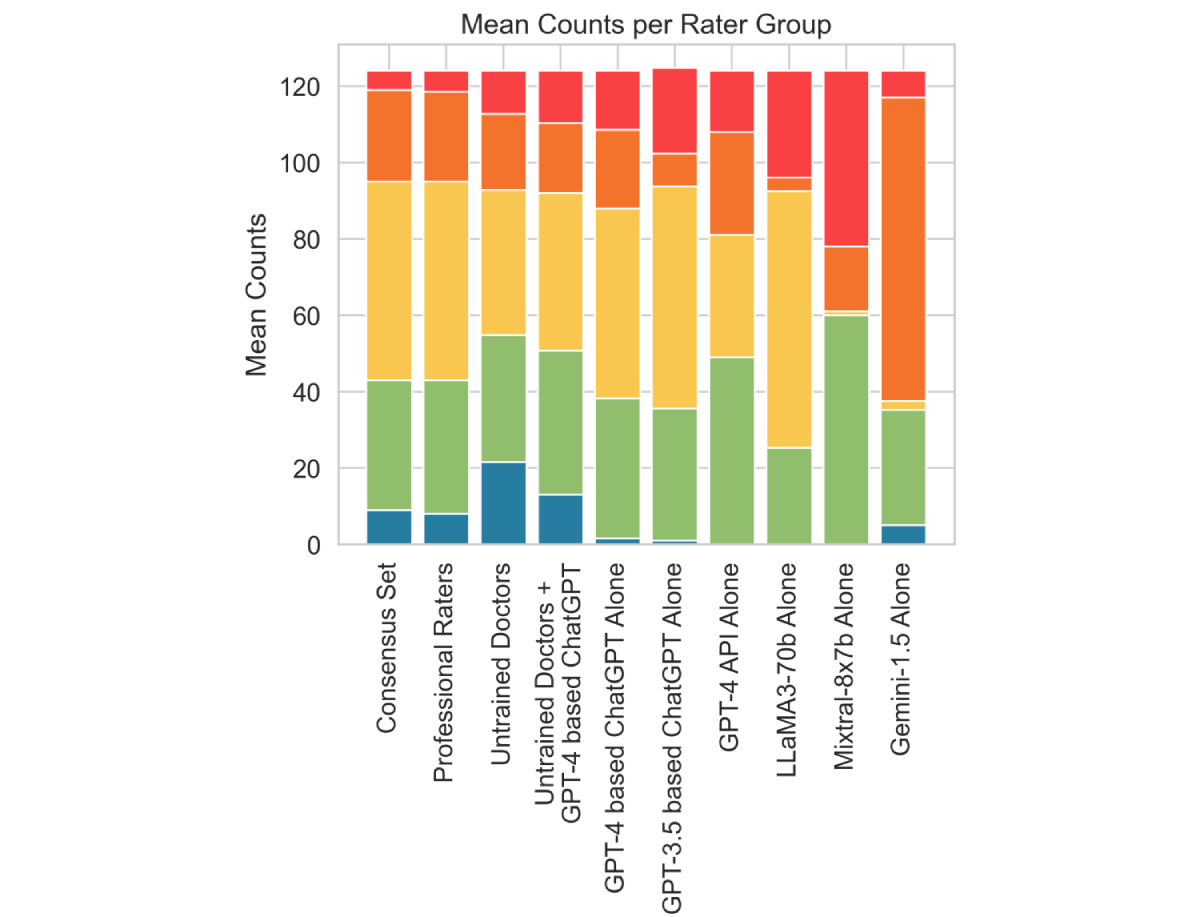
Average distribution of Manchester Triage System (MTS) levels among different rater groups. The figure presents stacked bar plots illustrating the average count of triage categories, arranged in ascending order of severity. The least severe level, Level 5 ("Blue"), is located at the bottom, while the most severe level, Level 1 ("Red"), is positioned at the top. API: application programming interface.

In addition to the overall agreement with the consensus set, the distribution of assigned MTS levels varied considerably among the different rater groups. In comparison to professional raters and the consensus set, ChatGPT and other LLMs except for Gemini 1.5 frequently assigned the highest triage category “Red” (mean 18.1%, SD 0.5% GPT-3.5–based ChatGPT; mean 12.5%, SD 2.5% GPT-4–based ChatGPT; mean 22.5%, SD 0.7% Llama 3 70B; mean 37.1%, SD 0% Mixtral 8x7b; 5/124, 4% consensus set), yet they seldomly or never designated the lowest category “Blue” (only once across 4 batches with 124 questions [mean 0.2%] for GPT-3.5–based ChatGPT; mean 1.2%, SD 0.5% GPT-4–based ChatGPT; 9/124, 7.3% consensus set; Llama 3 70B, raw GPT-4, and Mixtral 7x8b never assigned the category). As shown, this pattern was more evident in GPT-3.5–based ChatGPT than in version 4 and less evident in Gemini 1.5 (mean 5.6%, SD 0% “Red” and mean 4%, SD 0% “Blue”). In contrast, untrained doctors assigned the “Blue” category much more frequently than their professionally trained counterparts (mean 17.3%, SD 8.4% untrained doctors vs 9/124, 7.3% consensus set), while also employing the “Red” category more often (mean 9.1%, SD 5.5% untrained doctors vs 5/124, 4% consensus set). Certain language models displayed notable triaging patterns, with Gemini 1.5 predominantly categorizing cases as “Orange” (mean 64.1%, SD1.1% Gemini 1.5 vs 24/124, 19.4% consensus set), while Mixtral 7x8b frequently selected “Red” (mean 37.1%, SD 0%) and “Green” (mean 48.4%, SD 0%) and yielded equal results over all 4 iterations (see Figure 3). Accordingly, tendencies toward overtriage and undertriage were evident, with the GPT models leaning toward overtriage, untrained doctors more often undertriaging, and the hybrid models displaying a fairly balanced performance (see Figure S4 in Multimedia Appendix 1). Among untrained doctors, variance in triage strategies was noticeable, as demonstrated by the composition of individual raters’ triages (see Figure S5 in Multimedia Appendix 1). Importantly, all raters and LLMs, except for 2 instances where untrained doctors rated a “Red” case as “Orange,” accurately identified the most critical “Red” cases in the consensus set as “Red.” Notably, 1 doctor corrected their initial misjudgment to “Red” after being provided the GPT assessment.

## Discussion

With the rising number of acute cases in the ED and ongoing limitations of personnel resources [16,17], strategies for improving triage are urgently needed to increase the capacity, motivation, and opportunities of nurses and doctors. Past attempts to decrease workload, such as patient self-triage, have shown limited correlation when compared to triages conducted by nurses trained in the MTS [33]. However, recent results from algorithmic solutions demonstrate their potential in enhancing clinical decision processes and optimizing resource utilization [34].

Given the swift advancements in natural language processing made by LLMs and their extensive applications in medical care, it is plausible that such models could further enhance ED operations in the near future. It is worth noting that in our scenario, no clarifying questions or similar interactions were allowed, which may not mirror the typical setting of an ED. A test of the consumer-facing, general LLM product ChatGPT appeared especially relevant, as many health care professionals have access to and experimented with this product. Our study findings suggest that even a general-purpose system such as ChatGPT can match the performance of untrained doctors when triaging emergency cases and, similar to previous models [35], consistently identify the most severe cases. Similar on-par performance between GPT-4–based ChatGPT and ophthalmology trainees has been reported in another medical context, further underlining this finding [24]. However, the findings clearly highlight that the tested versions of ChatGPT did not demonstrate gold-standard performance in triage, especially in more ambiguous cases, which is also in line with previous findings from a study from Turkey [23]. This study found varying results for other LLMs, with the performance of Gemini 1.5, Llama 3 70B, and Mixtral 8x7b being similar to or worse than the GPT-4–based ChatGPT or GPT-4 itself. Importantly, this research employed a zero-shot approach, suggesting that parameter tuning could not only enhance results but also address occasional irregular triaging behavior, which warrants sophisticated testing in future studies.

The integration of LLMs in ED triage is promising not only in terms of decision accuracy but also in potentially boosting efficiency and saving time, a critical factor in emergency scenarios. However, it should be pointed out that this study does not empirically measure their efficiency against traditional triage methods. Future studies are needed to rigorously evaluate the time and effort implications of integrating LLMs and AI assistants into clinical settings, particularly in terms of data entry requirements and comparison with established triage practices by trained medical staff. Such evaluations should incorporate interdisciplinary approaches including nursing staff, who are often responsible for triage in the ED.

Despite the considerable potential of using such a system as a second-opinion tool for clinicians—as demonstrated in our study—the minimal actual improvement observed indicates an ongoing need to train medical personnel in more efficient usage of these resources [36]. While 1 untrained rater corrected an undertriage to the most critical color “Red” after seeing the ChatGPT ratings—a change that could be clinically significant—further studies are needed to explore potential quality improvements with better validated systems and professionally trained triage raters.

The substantial progress between ChatGPT using GPT-3.5 and GPT-4—performing comparably to untrained doctors—hints at further potential advancements in this area. Since a very basic prompting strategy without background on the copyright-protected MTS or strategies such as retrieval-augmented generation was used, there appears to be ample room for further technical improvements. Given the option to fine-tune and further train LLMs on specific data, such as triage cases, it is well conceivable that they might even yield better results that are on par with professional raters in the future and take a wider range of parameters into account than humans are able to. Furthermore, these systems could help decrease the workload of qualified medical staff by answering basic inquiries, collecting medical information, and offering further differential diagnoses as previously shown [37]. When combined with the recent advancements in automatic patient decompensation detection using continuous sensor data in EDs [38], these technologies could contribute to an efficient and human-centered ED experience for both staff and patients. Moreover, these systems might, in the future, assist patients in deciding between primary care and ED presentation. However, such a prediction warrants further investigation.

It is crucial to note that this study is purely exploratory, and at present, no clinical decisions should be based solely on recommendations made by an LLM or ChatGPT without proper validation studies [39] in place. This is further underscored by the LLMs’ and ChatGPT’s performance deficiencies in this study, which would likely lead to suboptimal triage if used in a real-world setting. While this research emphasizes the potential and rapid progress of such models in health care and EDs, the authors want to highlight the ongoing debate about the appropriate regulation of these models for medical applications and how data privacy concerns should be addressed [40,41]. Notwithstanding these challenges, as researchers diligently navigate and promote responsible innovation, the potential of AI to revolutionize health care and augment patient outcomes perseveres as a captivating opportunity to contribute to the amelioration of our health care system. Future validation studies should further prioritize large, representative, and multicentric data sets, which are paramount for the correct assessment of AI tools. In summary, despite rapid advancements in LLM technologies and associated products such as ChatGPT, this study confirms that in a very basic setup, they currently do not meet the gold standard for ED triage, underscoring the urgent need for further development and rigorous validation.

## Appendix

Multimedia Appendix 1 Supplementary material.

## Abbreviations

AI: artificial intelligence
GPT: Generative Pre-trained Transformer
ED: emergency department
LLM: large language model
MTS: Manchester Triage System

## Data Availability

The data assessed in this study are available from the corresponding author on reasonable request.

## References

[1] Al-Zaiti SS, Martin-Gill C, Zègre-Hemsey JK, Bouzid Z, Faramand Z, Alrawashdeh MO, Gregg RE, Helman S, Riek NT, Kraevsky-Phillips K, Clermont G, Akcakaya M, Sereika SM, van Dam P, Smith SW, Birnbaum Y, Saba S, Sejdic E, Callaway CW (2023). Machine learning for ECG diagnosis and risk stratification of occlusion myocardial infarction. Nat Med.

[2] Smak Gregoor AM, Sangers TE, Bakker LJ, Hollestein L, Uyl-de Groot CA, Nijsten T, Wakkee M (2023). An artificial intelligence based app for skin cancer detection evaluated in a population based setting. NPJ Digit Med.

[3] Shin HJ, Han K, Ryu L, Kim E (2023). The impact of artificial intelligence on the reading times of radiologists for chest radiographs. NPJ Digit Med.

[4] (2022). Introducing ChatGPT. OpenAI.

[5] Kung TH, Cheatham M, Medenilla A, Sillos C, De Leon L, Elepaño C, Madriaga M, Aggabao R, Diaz-Candido G, Maningo J, Tseng V (2023). Performance of ChatGPT on USMLE: potential for AI-assisted medical education using large language models. PLOS Digit Health.

[6] Jung LB, Gudera JA, Wiegand TLT, Allmendinger S, Dimitriadis K, Koerte IK (2023). ChatGPT passes German state examination in medicine with picture questions omitted. Dtsch Arztebl Int.

[7] Ayers JW, Poliak A, Dredze M, Leas EC, Zhu Z, Kelley JB, Faix DJ, Goodman AM, Longhurst CA, Hogarth M, Smith DM (2023). Comparing physician and artificial intelligence chatbot responses to patient questions posted to a public social media forum. JAMA Intern Med.

[8] Sarraju A, Bruemmer D, Van Iterson E, Cho L, Rodriguez F, Laffin L (2023). Appropriateness of cardiovascular disease prevention recommendations obtained from a popular online chat-based artificial intelligence model. JAMA.

[9] Patel SB, Lam K (2023). ChatGPT: the future of discharge summaries?. Lancet Digit Health.

[10] Gao CA, Howard FM, Markov NS, Dyer EC, Ramesh S, Luo Y, Pearson AT (2023). Comparing scientific abstracts generated by ChatGPT to real abstracts with detectors and blinded human reviewers. NPJ Digit Med.

[11] Nabla.

[12] Tierney AA, Gayre G, Hoberman B, Mattern B, Ballesca M, Kipnis P, Liu V, Lee K (2024). Ambient artificial intelligence scribes to alleviate the burden of clinical documentation. NEJM Catal Innov Care Deliv.

[13] McDuff D, Schaekermann M, Tu T, Palepu A, Wang A, Garrison J, Singhal K, Sharma Y, Azizi S, Kulkarni K, Hou L, Cheng Y, Liu Y, Mahdavi S, Prakash S, Pathak A, Semturs C, Patel S, Webster D, Dominowska E, Gottweis J, Barral J, Chou K, Corrado G, Matias Y, Sunshine J, Karthikesalingam A, Natarajan V Towards accurate differential diagnosis with large language models. arXiv.

[14] Gräff I, Goldschmidt B, Glien P, Bogdanow M, Fimmers R, Hoeft A, Kim S, Grigutsch D (2014). The German version of the Manchester Triage System and its quality criteria--first assessment of validity and reliability. PLoS One.

[15] Gräff I, Latzel B, Glien P, Fimmers R, Dolscheid-Pommerich RC (2019). Validity of the Manchester Triage System in emergency patients receiving life-saving intervention or acute medical treatment-a prospective observational study in the emergency department. J Eval Clin Pract.

[16] Healy S, Tyrrell M (2011). Stress in emergency departments: experiences of nurses and doctors. Emerg Nurse.

[17] García-Tudela Á, Simonelli-Muñoz AJ, Rivera-Caravaca JM, Fortea MI, Simón-Sánchez Lucas, González-Moro MTR, González-Moro JMR, Jiménez-Rodríguez D, Gallego-Gómez JI (2022). Stress in emergency healthcare professionals: the Stress Factors and Manifestations Scale. Int J Environ Res Public Health.

[18] Zachariasse JM, Seiger N, Rood PPM, Alves CF, Freitas P, Smit FJ, Roukema GR, Moll HA (2017). Validity of the Manchester Triage System in emergency care: a prospective observational study. PLoS One.

[19] Fekonja Z, Kmetec S, Fekonja U, Mlinar Reljić N, Pajnkihar M, Strnad M (2023). Factors contributing to patient safety during triage process in the emergency department: a systematic review. J Clin Nurs.

[20] Gemini 1.5. Google.

[21] Llama 3. Meta.

[22] Mixtral AI.

[23] Sarbay İ, Berikol GB, Özturan İU (2023). Performance of emergency triage prediction of an open access natural language processing based chatbot application (ChatGPT): a preliminary, scenario-based cross-sectional study. Turk J Emerg Med.

[24] Lyons RJ, Arepalli SR, Fromal O, Choi JD, Jain N (2023). Artificial intelligence chatbot performance in triage of ophthalmic conditions. Can J Ophthalmol.

[25] Mackway-Jones K, Marsden J, Windle J, Krey J, Moecke H (2020). Ersteinschätzung in der Notaufnahme - Das Manchester-Triage-System. 5th ed.

[26] Perlis RH, Fihn SD (2023). Evaluating the application of large language models in clinical research contexts. JAMA Netw Open.

[27] Cohen J (1960). A coefficient of agreement for nominal scales. Educ Psychol Meas.

[28] Seabold S, Perktold J, van der Walt S, Millman J (2010). Statsmodels: econometric and statistical modeling with Python. Proceedings of the 9th Python in Science Conference (SciPy 2010).

[29] Hunter JD (2007). Matplotlib: a 2D graphics environment. Comput Sci Eng.

[30] Waskom ML (2021). seaborn: statistical data visualization. Journal of Open Source Software.

[31] Michael M, Al Agha S, Böhm L, Bosse HM, Pohle AN, Schürmann J, Hannappel O, Tengg E, Weiß C, Bernhard M (2023). Alters- und geschlechtsbezogene Verteilung von Zuführung, Ersteinschätzung, Entlassart und Verweildauer in der zentralen Notaufnahme [Article in German]. Notfall Rettungsmed.

[32] Olofsson P, Gellerstedt M, Carlström Eric D (2009). Manchester Triage in Sweden - interrater reliability and accuracy. Int Emerg Nurs.

[33] Dickson SJ, Dewar C, Richardson A, Hunter A, Searle S, Hodgson LE (2022). Agreement and validity of electronic patient self-triage (eTriage) with nurse triage in two UK emergency departments: a retrospective study. Eur J Emerg Med.

[34] Boonstra A, Laven M (2022). Influence of artificial intelligence on the work design of emergency department clinicians a systematic literature review. BMC Health Serv Res.

[35] Raita Y, Goto T, Faridi MK, Brown DFM, Camargo CA, Hasegawa K (2019). Emergency department triage prediction of clinical outcomes using machine learning models. Crit Care.

[36] Paranjape K, Schinkel M, Nannan Panday R, Car J, Nanayakkara P (2019). Introducing artificial intelligence training in medical education. JMIR Med Educ.

[37] Hirosawa T, Harada Y, Yokose M, Sakamoto T, Kawamura R, Shimizu T (2023). Diagnostic accuracy of differential-diagnosis lists generated by Generative Pretrained Transformer 3 chatbot for clinical vignettes with common chief complaints: a pilot study. Int J Environ Res Public Health.

[38] Sundrani S, Chen J, Jin BT, Abad ZSH, Rajpurkar P, Kim D (2023). Predicting patient decompensation from continuous physiologic monitoring in the emergency department. NPJ Digit Med.

[39] Goldsack JC, Coravos A, Bakker JP, Bent B, Dowling AV, Fitzer-Attas C, Godfrey A, Godino JG, Gujar N, Izmailova E, Manta C, Peterson B, Vandendriessche B, Wood WA, Wang KW, Dunn J (2020). Verification, analytical validation, and clinical validation (V3): the foundation of determining fit-for-purpose for biometric monitoring technologies (BioMeTs). NPJ Digit Med.

[40] Minssen T, Vayena E, Cohen IG (2023). The challenges for regulating medical use of ChatGPT and other large language models. JAMA.

[41] Meskó B, Topol EJ (2023). The imperative for regulatory oversight of large language models (or generative AI) in healthcare. NPJ Digit Med.

