# Supplementary material for: Triage Performance Across Large Language Models, ChatGPT, and Untrained Doctors in Emergency Medicine: Comparative Study
Source: J Med Internet Res. 2024 Jun 14;26:e53297. doi: 10.2196/53297 (PMC11214027; doi:10.2196/53297)
Supplement: Multimedia Appendix 1 [file jmir_v26i1e53297_app1.pdf]

Multimedia Appendix 1 Overview

Ordered by appearance in main text

1. Textbox S1: Example Case Vignettes
2. Textbox S2: Prompt used for the Generation of LLM Triages
3. Textbox S3: Example ChatGPT Output (for Example Case Vignette 1) used as Second Opinion.
4. Table S1: Large Language Models and Respective Settings
5. Figure S1: Triage Results per individual Rater (Doctors and ChatGPT).
6. Figure S2: Triage Results per individual Rater (Other LLMs).
7. Table S2: Results of Tukey's Honest Significant Difference Test
8. Figure S3: Quadratic Weighted Cohen’s Kappa Agreement for Individual Raters and Three Scenarios
9. Figure S4: Overtriage and Undertriage Rate per Group and Individual Rater
10. Figure S5: Average Distribution of Manchester Triage System Levels per Individual Rater

1. *“A 36-year-old female walks into the ER at 3 am. She recently renovated her apartment and is sleeping there for the first time. She has experienced increasing allergic asthma over the past few weeks. Currently, she's coughing with facial redness. Shortly after arrival, she exhibits dyspnea and inspiratory stridor.”*

2. *“A 27-year-old male patient reports increased ear pain on his right side following a respiratory infection last week. He took an unspecified antibiotic until yesterday. He has a mild hearing impairment in his right ear but no nausea, dizziness, or otorrhoea. He doesn't have a fever or any neurological deficits. He rates the pain as 5/10 on the NAS scale.*

3. *“A 68-year-old female patient walks in with severe left-sided pain (NAS 10/10). Has a history of kidney stones, and describes the current episode to be very similar. Currently hypertensive (180mmHg sys), other vitals normal. No other symptoms, no other relevant medical history. No immunosuppression or international journeys.”*

Textbox S1: Example Case Vignettes

Prompt used for ChatGPT: *“I would like to test your ability to perform an emergency room triage according to the Manchester Triage System. I will present you several cases. The provided information is all you get. If there is something missing, please assume it is not relevant to the case. As an answer, please tell me the triage color and very briefly explain your decision.”*

Prompt used for APIs: *“You are a professional medical chatbot. I would like to test your ability to perform an emergency room triage according to the Manchester Triage System. The provided information is all you get. If there is something missing, please assume it is not relevant to the case. As an answer, please tell me the triage color and very briefly explain your decision. Give a brief answer. Only return the triage color and the reasoning. [Followed by newline and case].”*

Textbox S2: Prompt used for the Generation of LLM Triages

“Triage Color: Red

The patient is presenting with severe respiratory distress, indicated by dyspnea (difficulty breathing) and inspiratory stridor (a high-pitched, wheezing sound due to obstructed air flow). These symptoms suggest she may be experiencing a severe asthma attack or possibly an allergic reaction that's causing her airways to tighten. Immediate medical intervention is needed to open her airways and normalize her breathing.”

Textbox S3: Example ChatGPT Output (for Example Case Vignette 1) used as Second Opinion.

	GPT-4	Gemini-1.5	LLaMA3-70b	Mixtral-8x7b
**Exact version**	gpt-4-1106-preview	gemini-1.5-pro-preview-0409	llama3-70b-8192	mixtral-8x7b-32768
**API**	Openai	Google Cloud	Groq.com	Groq.com
**Temperature**	0	0	0	0
**Top_p**	0.95	0.95	0.95	0.95
**Maximal tokens**	300	300	300	300
**Other settings**	Default	Safety settings (HARM_CATEGORY_HATE_SPEECH, HARM_CATEGORY_DANGEROUS_CONTENT, HARM_CATEGORY_SEXUALLY_EXPLICIT, HARM_CATEGORY_HARASSMENT) all set to BLOCK_ONLY_HIGH	Default	Default

Table S1: Large Language Models and Respective Settings

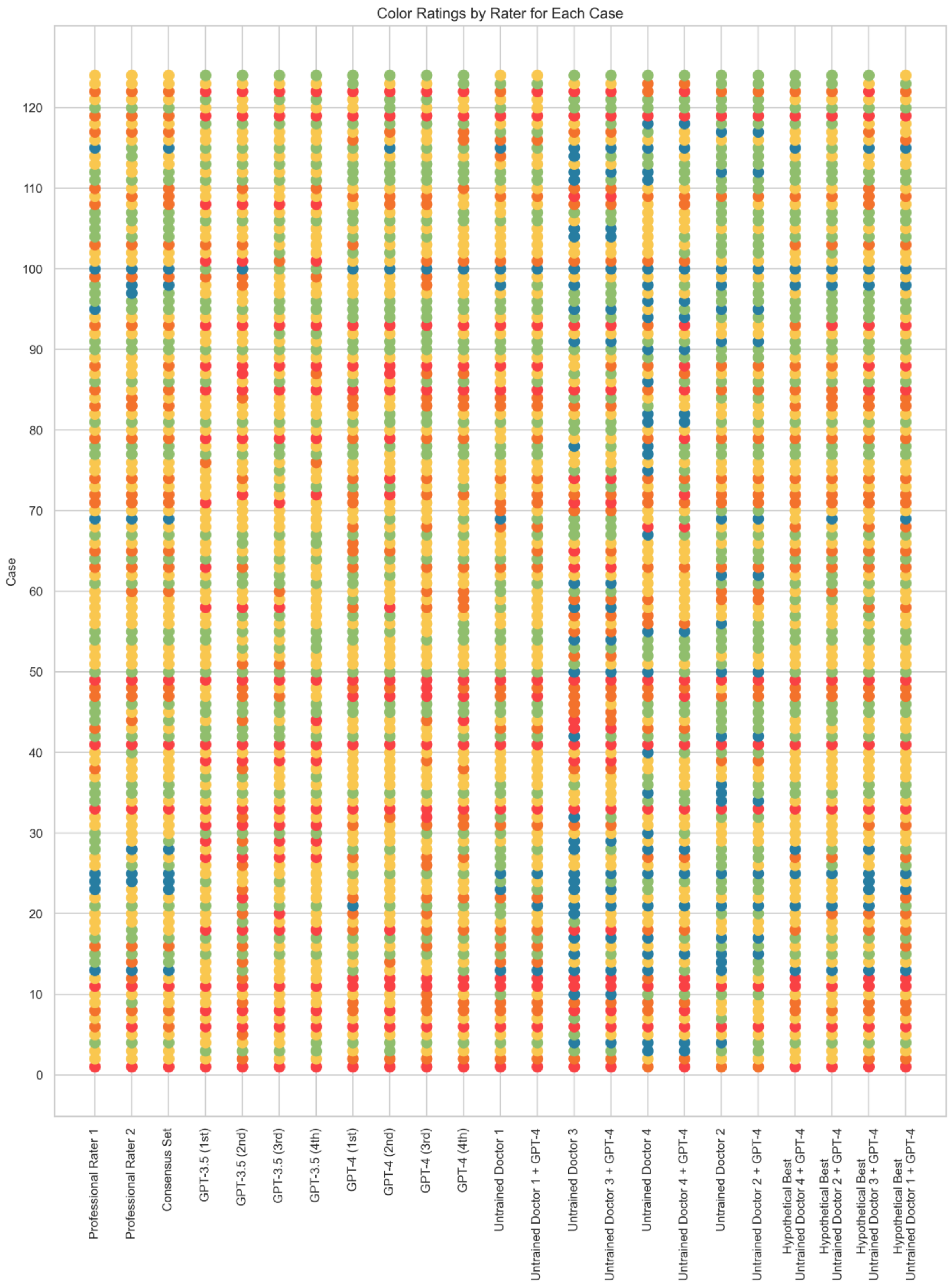
Color Ratings by Rater for Each Case
Case
0
10
20
30
40
50
60
70
80
90
100
110
120
Professional Rater 1
Professional Rater 2
Consensus Set
GPT-3.5 (1st)
GPT-3.5 (2nd)
GPT-3.5 (3rd)
GPT-3.5 (4th)
GPT-4 (1st)
GPT-4 (2nd)
GPT-4 (3rd)
GPT-4 (4th)
Untrained Doctor 1
Untrained Doctor 1 + GPT-4
Untrained Doctor 3
Untrained Doctor 3 + GPT-4
Untrained Doctor 4
Untrained Doctor 4 + GPT-4
Untrained Doctor 2
Untrained Doctor 2 + GPT-4
Hypothetical Best Untrained Doctor 4 + GPT-4
Hypothetical Best Untrained Doctor 2 + GPT-4
Hypothetical Best Untrained Doctor 3 + GPT-4
Hypothetical Best Untrained Doctor 1 + GPT-4

Figure S1: Triage Results per individual Rater (Doctors and ChatGPT versions). This plot shows for each triage case the individual assessment per rater. On the y-axis the case number and on the x-axis the individual raters are shown. The color code represents the triage class according to the Manchester Triage levels from level 1 'Red' – immediate assessment – to level 5 'Blue' - non-urgent assessment. GPT-4 and GPT-3.5 refer to GPT-4 based ChatGPT and GPT-3.5 based ChatGPT accordingly.

Figure S2: Triage Results per individual Rater (Other LLMs).
This plot shows for each triage case the individual assessment per large language model (LLM) queried via application programming interface. The consensus set is displayed as reference on the very left. On the y-axis the case number and on the x-axis the individual raters are shown. The color code represents the triage class according to the Manchester Triage levels from level 1 'Red' – immediate assessment – to level 5 'Blue' - non-urgent assessment.

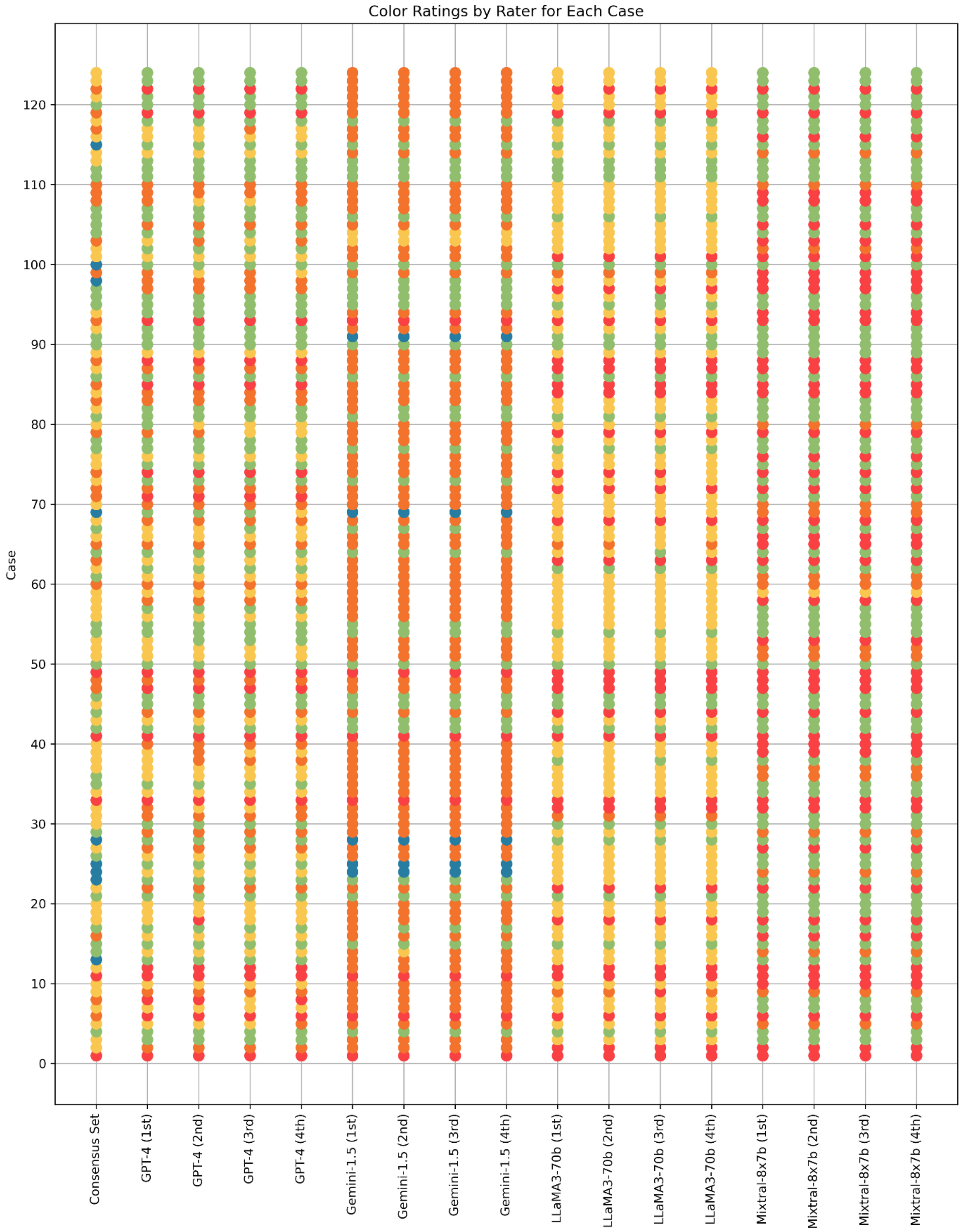
Color Ratings by Rater for Each Case
Case
0
10
20
30
40
50
60
70
80
90
100
110
120
Consensus Set
GPT-4 (1st)
GPT-4 (2nd)
GPT-4 (3rd)
GPT-4 (4th)
Gemini-1.5 (1st)
Gemini-1.5 (2nd)
Gemini-1.5 (3rd)
Gemini-1.5 (4th)
LLaMA3-70b (1st)
LLaMA3-70b (2nd)
LLaMA3-70b (3rd)
LLaMA3-70b (4th)
Mixtral-8x7b (1st)
Mixtral-8x7b (2nd)
Mixtral-8x7b (3rd)
Mixtral-8x7b (4th)

Group 1	Group 2	Mean Difference	p-adjusted	Lower 95% CI	Upper 95% CI
GPT-3.5 ChatGPT Alone	GPT-4 API Alone	0.1031	**0.0031**	0.0266	0.1796
GPT-3.5 ChatGPT Alone	GPT-4 ChatGPT Alone	0.127	**0.0002**	0.0504	0.2035
GPT-3.5 ChatGPT Alone	Gemini-1.5 Alone	0.056	0.2888	-0.0205	0.1325
GPT-3.5 ChatGPT Alone	LLaMA3-70b Alone	-0.0236	0.9773	-0.1001	0.053
GPT-3.5 ChatGPT Alone	Mixtral-8x7b Alone	-0.1209	**0.0004**	-0.1974	-0.0443
GPT-3.5 ChatGPT Alone	Professional Raters	0.3693	**< 0.0001**	0.2756	0.463
GPT-3.5 ChatGPT Alone	Untrained Doctors	0.1365	**0.0001**	0.06	0.213
GPT-3.5 ChatGPT Alone	Untrained Doctors + GPT-4	0.1615	**< 0.0001**	0.085	0.238
GPT-4 API Alone	GPT-4 ChatGPT Alone	0.0239	0.9754	-0.0526	0.1004
GPT-4 API Alone	Gemini-1.5 Alone	-0.0471	0.5043	-0.1236	0.0294
GPT-4 API Alone	LLaMA3-70b Alone	-0.1266	**0.0002**	-0.2031	-0.0501
GPT-4 API Alone	Mixtral-8x7b Alone	-0.2239	**< 0.0001**	-0.3004	-0.1474
GPT-4 API Alone	Professional Raters	0.2662	**< 0.0001**	0.1725	0.3599
GPT-4 API Alone	Untrained Doctors	0.0334	0.8549	-0.0431	0.1099
GPT-4 API Alone	Untrained Doctors + GPT-4	0.0584	0.2414	-0.0181	0.1349
GPT-4 ChatGPT Alone	Gemini-1.5 Alone	-0.071	0.0842	-0.1475	0.0055
GPT-4 ChatGPT Alone	LLaMA3-70b Alone	-0.1505	**< 0.0001**	-0.227	-0.074
GPT-4 ChatGPT Alone	Mixtral-8x7b Alone	-0.2478	**< 0.0001**	-0.3243	-0.1713
GPT-4 ChatGPT Alone	Professional Raters	0.2423	**< 0.0001**	0.1487	0.336
GPT-4 ChatGPT Alone	Untrained Doctors	0.0095	1	-0.067	0.086
GPT-4 ChatGPT Alone	Untrained Doctors + GPT-4	0.0345	0.8314	-0.042	0.1111
Gemini-1.5 Alone	LLaMA3-70b Alone	-0.0795	**0.0372**	-0.156	-0.003
Gemini-1.5 Alone	Mixtral-8x7b Alone	-0.1768	**< 0.0001**	-0.2533	-0.1003

Gemini-1.5 Alone	Professional Raters	0.3133	**< 0.0001**	0.2196	0.407
Gemini-1.5 Alone	Untrained Doctors	0.0805	**0.0338**	0.004	0.157
Gemini-1.5 Alone	Untrained Doctors + GPT-4	0.1055	**0.0024**	0.029	0.182
LLaMA3-70b Alone	Mixtral-8x7b Alone	-0.0973	**0.0058**	-0.1738	-0.0208
LLaMA3-70b Alone	Professional Raters	0.3929	**< 0.0001**	0.2992	0.4865
LLaMA3-70b Alone	Untrained Doctors	0.16	**< 0.0001**	0.0835	0.2365
LLaMA3-70b Alone	Untrained Doctors + GPT-4	0.185	**< 0.0001**	0.1085	0.2616
Mixtral-8x7b Alone	Professional Raters	0.4902	**< 0.0001**	0.3965	0.5838
Mixtral-8x7b Alone	Untrained Doctors	0.2573	**< 0.0001**	0.1808	0.3338
Mixtral-8x7b Alone	Untrained Doctors + GPT-4	0.2823	**< 0.0001**	0.2058	0.3589
Professional Raters	Untrained Doctors	-0.2328	**< 0.0001**	-0.3265	-0.1391
Professional Raters	Untrained Doctors + GPT-4	-0.2078	**< 0.0001**	-0.3015	-0.1141
Untrained Doctors	Untrained Doctors + GPT-4	0.025	0.9675	-0.0515	0.1015

Table S2: Results of Tukey's Honest Significant Difference Test

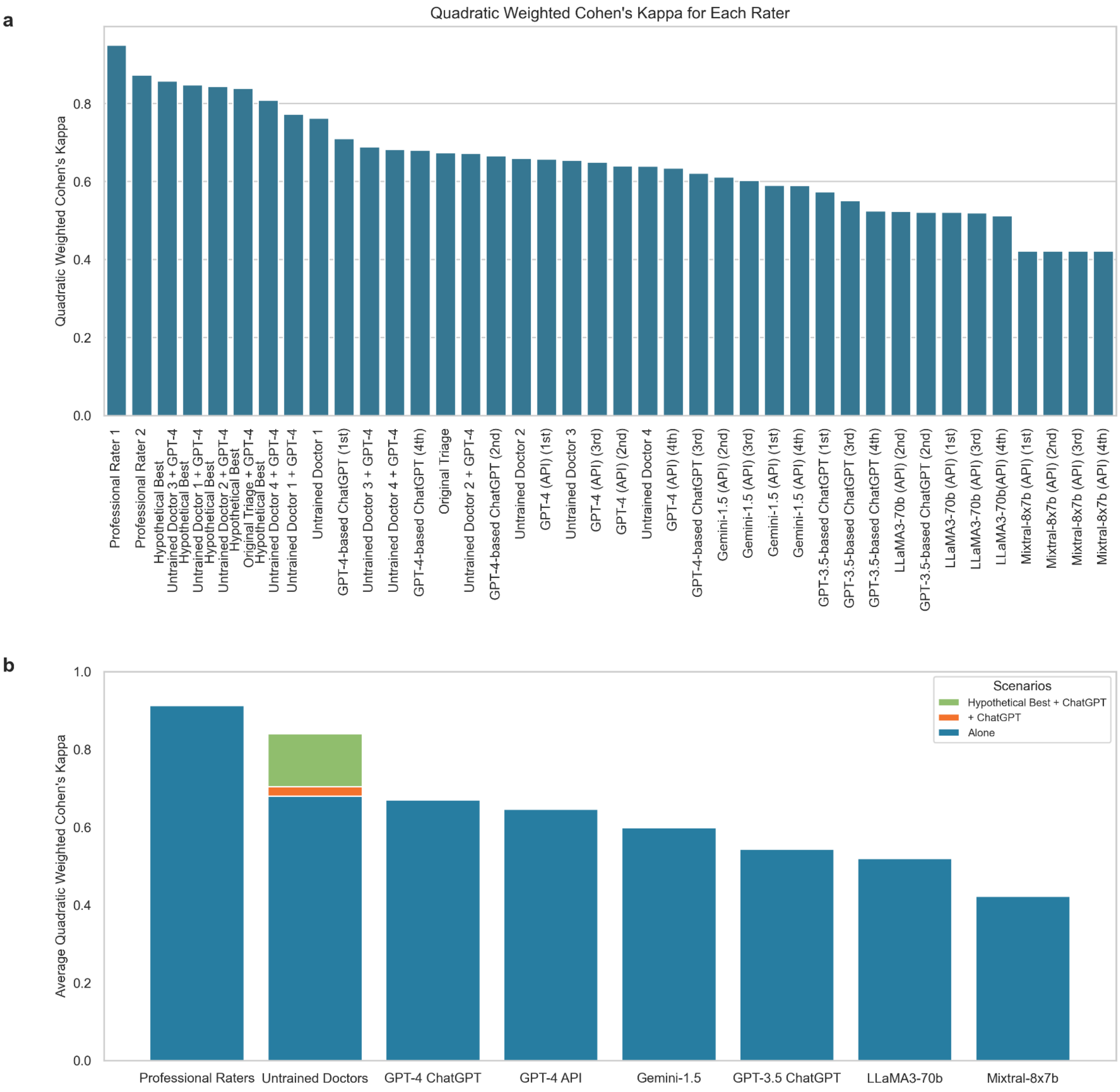

Figure S3: Quadratic Weighted Cohen's Kappa Agreement for Individual Raters and Three Scenarios.

a. The bar chart presents the quadratic weighted Cohen's Kappa scores for each individual rater (doctor or large language model) and the performance of untrained doctors utilizing GPT-4 based ChatGPT for a second opinion. Additionally, the maximal performance for untrained doctors using

hypothetically the best possible combination of GPT-4 based ChatGPT responses and their own assessment is shown.

b. This bar chart uses the average quadratic weighted Cohen's kappa values for the two ChatGPT Groups (GPT-4 based or GPT-3.5 based), the different other queried large language models and the untrained doctors (blue). Improved performances for untrained doctors using GPT-4 based ChatGPT as second opinion (orange), as well as the hypothetical best possible score merging their assessments (green) are depicted.

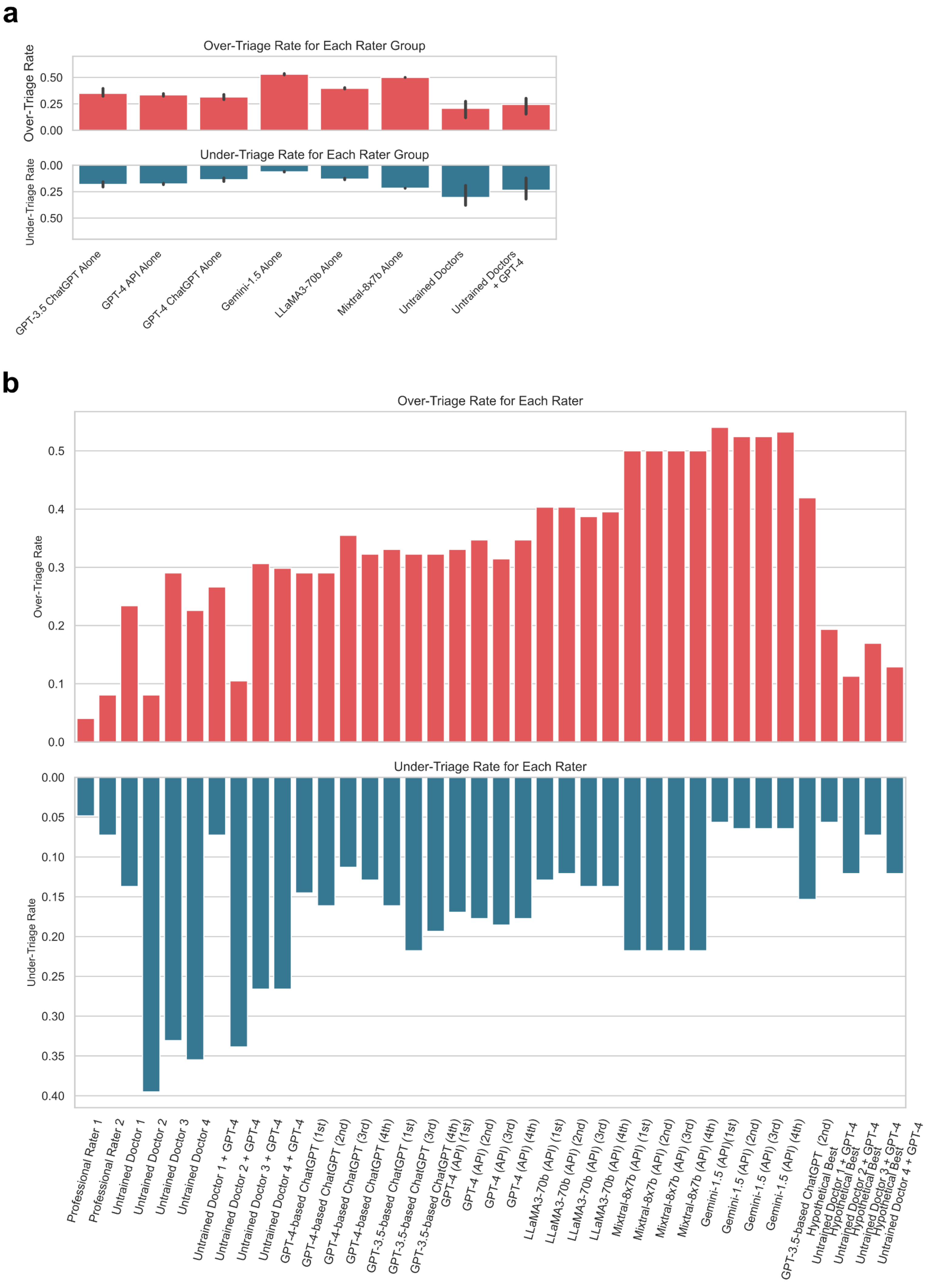
a
Over-Triage Rate for Each Rater Group
Over-Triage Rate
0.50
0.25
0.00
Under-Triage Rate for Each Rater Group
Under-Triage Rate
0.00
0.25
0.50
GPT-3.5 ChatGPT Alone
GPT-4 API Alone
GPT-4 ChatGPT Alone
Gemini-1.5 Alone
LLaMA3-70b Alone
Mixtral-8x7b Alone
Untrained Doctors
Untrained Doctors + GPT-4
b
Over-Triage Rate for Each Rater
Over-Triage Rate
0.5
0.4
0.3
0.2
0.1
0.0
Under-Triage Rate for Each Rater
Under-Triage Rate
0.00
0.05
0.10
0.15
0.20
0.25
0.30
0.35
0.40
Professional Rater 1
Professional Rater 2
Untrained Doctor 1
Untrained Doctor 2
Untrained Doctor 3
Untrained Doctor 4
Untrained Doctor 1 + GPT-4
Untrained Doctor 2 + GPT-4
Untrained Doctor 3 + GPT-4
Untrained Doctor 4 + GPT-4
GPT-4-based ChatGPT (1st)
GPT-4-based ChatGPT (2nd)
GPT-4-based ChatGPT (3rd)
GPT-4-based ChatGPT (4th)
GPT-3.5-based ChatGPT (1st)
GPT-3.5-based ChatGPT (3rd)
GPT-3.5-based ChatGPT (4th)
GPT-4 (API) (1st)
GPT-4 (API) (2nd)
GPT-4 (API) (3rd)
GPT-4 (API) (4th)
LLaMA3-70b (API) (1st)
LLaMA3-70b (API) (2nd)
LLaMA3-70b (API) (3rd)
LLaMA3-70b (API) (4th)
Mixtral-8x7b (API) (1st)
Mixtral-8x7b (API) (2nd)
Mixtral-8x7b (API) (3rd)
Mixtral-8x7b (API) (4th)
Gemini-1.5 (API)(1st)
Gemini-1.5 (API) (2nd)
Gemini-1.5 (API) (3rd)
Gemini-1.5 (API) (4th)
GPT-3.5-based ChatGPT (2nd)
Hypothetical Best
Untrained Doctor 1 + GPT-4
Hypothetical Best
Untrained Doctor 2 + GPT-4
Hypothetical Best
Untrained Doctor 3 + GPT-4
Hypothetical Best
Untrained Doctor 4 + GPT-4

Figure S4: Overtriage and Undertriage Rate per Group and Individual Rater.

a. The upper bar chart presents the percentage of overtriaged cases for each rater group and the corresponding standard deviation among the group. Accordingly, the lower chart shows the percentage of undertriage cases.

b. The bar charts present similar to a) the undertriage and overtriage rate, but for each individual rater.

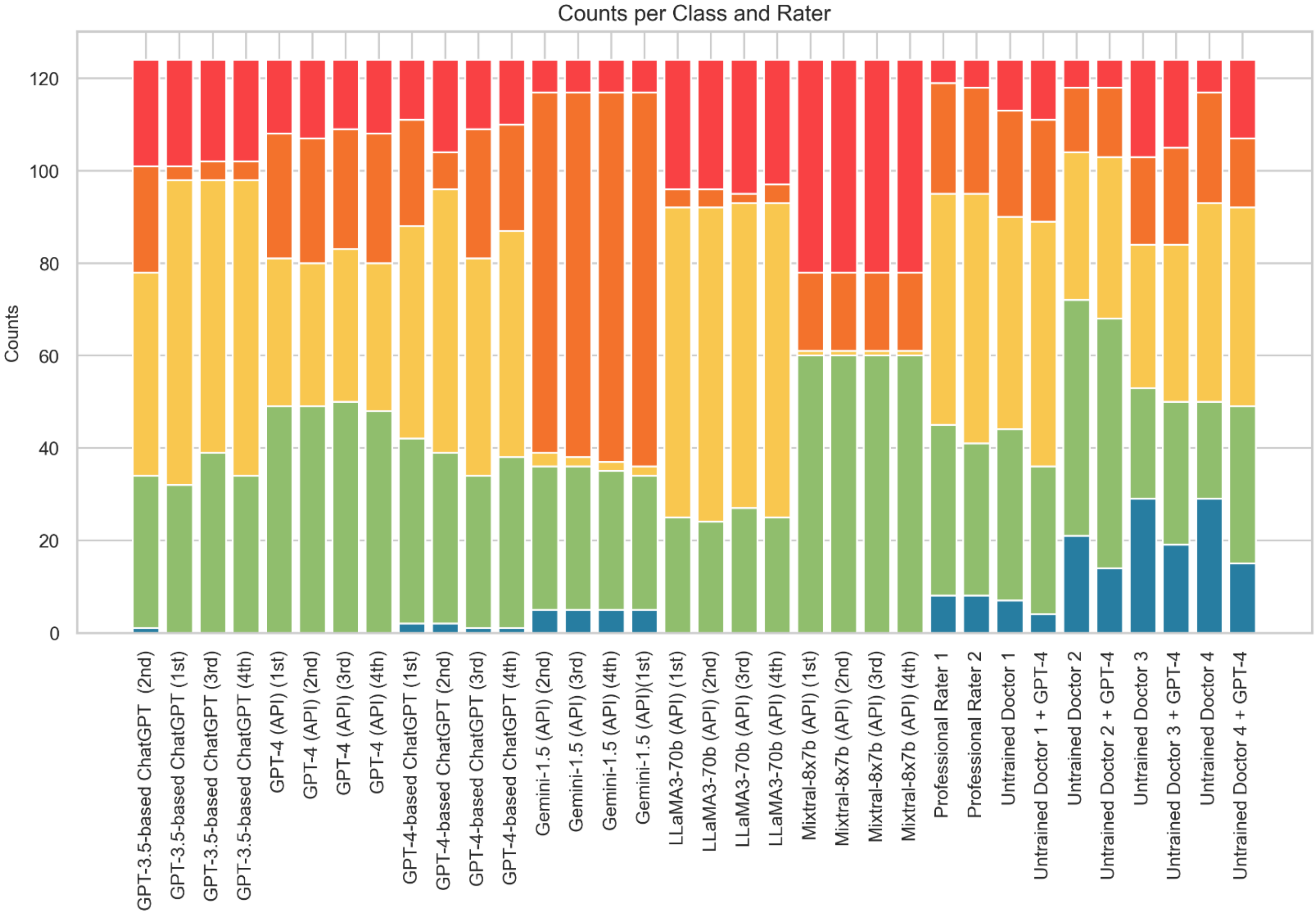

Figure S5: Average Distribution of Manchester Triage System Levels per Individual Rater.

The figure presents stacked bar plots illustrating the average count of triage categories according to the Manchester Triage System, arranged in ascending order of severity. The least severe level, Level 5 ('Blue'), is located at the bottom, while the most severe level, Level 1 ('Red'), is positioned at the top.
